# Deep sequencing analysis of toad *Rhinella schneideri* skin glands and partial biochemical characterization of its cutaneous secretion

**DOI:** 10.1186/s40409-018-0173-8

**Published:** 2018-11-29

**Authors:** Priscila Yumi Tanaka Shibao, Camila Takeno Cologna, Romualdo Morandi-Filho, Gisele Adriano Wiezel, Patricia Tiemi Fujimura, Carlos Ueira-Vieira, Eliane Candiani Arantes

**Affiliations:** 1 Laboratory of Animal Toxins, School of Pharmaceutical Scienes of Ribeirão Preto, University of São Paulo, Avenida do Café s/n, Ribeirão Preto, SP Brazil; 2 Laboratory of genetics – LABGEN, Institute of Genetics and Biochemistry, Campus Umuarama, Federal University of Uberlândia, Avenida Pará, Uberlândia, MG 1720 Brazil; 3 Department of Physics and Chemistry, University of São Paulo, School of Pharmaceutical Sciences of Ribeirão Preto, Av. do Café s/n°, Monte Alegre, Ribeirão Preto, SP 14040-903 Brazil

**Keywords:** RNA-seq, *Rhinella schneideri*, Toad secretion, Transcriptome, Illumina, Cutaneous secretion, Skin secretion, Toad protein

## Abstract

**Background:**

Animal poisons and venoms are sources of biomolecules naturally selected. *Rhinella schneideri* toads are widespread in the whole Brazilian territory and they have poison glands and mucous gland. Recently, protein from toads’ secretion has gaining attention. Frog skin is widely known to present great number of host defense peptides and we hypothesize toads present them as well. In this study, we used a RNA-seq analysis from *R. schneideri* skin and biochemical tests with the gland secretion to unravel its protein molecules.

**Methods:**

Total RNA from the toad skin was extracted using TRizol reagent, sequenced in duplicate using Illumina Hiseq2500 in paired end analysis. The raw reads were trimmed and de novo assembled using Trinity. The resulting sequences were submitted to functional annotation against non-redundant NCBI database and Database of Anuran Defense Peptide. Furthermore, we performed caseinolytic activity test to assess the presence of serine and metalloproteases in skin secretion and it was fractionated by fast liquid protein chromatography using a reverse-phase column. The fractions were partially sequenced by Edman’s degradation.

**Results:**

We were able to identify several classes of antimicrobial peptides, such as buforins, peroniins and brevinins, as well as PLA_2_, lectins and galectins, combining protein sequencing and RNA-seq analysis for the first time. In addition, we could isolate a PLA_2_ from the skin secretion and infer the presence of serine proteases in cutaneous secretion.

**Conclusions:**

We identified novel toxins and proteins from *R. schneideri* mucous glands. Besides, this is a pioneer study that presented the in depth characterization of protein molecules richness from this toad secretion. The results obtained herein showed evidence of novel AMP and enzymes that need to be further explored.

**Electronic supplementary material:**

The online version of this article (10.1186/s40409-018-0173-8) contains supplementary material, which is available to authorized users.

## Background

Animal’s and microorganisms’ secretions, as well as plant extracts, have been used as folk medicine since the dawn of the humanity [1].Therefore, molecules found in poisons and venoms are interesting, once they were selected by evolution to act in their molecular targets with high specificity [1, 2]. Such molecules can be used for feeding (predation), defense or even to dispose advantage in inter and intra-specific competition [3, 4]. As an example, the glandular secretory product from toads *Bufo melanostictus Schneider* and *Bufo Bufo gargarizans* cantor, known as Chan Su, is used as medicine in treatment for several physiological disturbances [5].

*Rhinella schneideri* toads are widely found in South America territory: Paraguay, Bolivia, Argentina, Uruguay and Brazil. Regarding the Brazilian territory, they are found especially in cerrado. These toads have shown remarkable adaptations skills and live in urban areas as well as rural [6].

These toads present two types of glands: granulous or parotoid and mucous glands. The first one is responsible for the animal protection against predators and are located in the postorbital region of the animal’s body; they can look bigger when the animal feels endangered due to body’s inflation, and acts as airbags against predator bites [7, 8]. The secretions are composed mainly by biogenic amines and steroids, as bufodienolides and bufotoxines, but they also produce proteins and glicoconjugate molecules [9, 10]. Although it was previously believed that this poison presented only few or even no protein, lately it was revealed this secretion possess up to 30% of weight in proteins, but there is a lack of data to full assess them [11, 12]. *Rhinella schneideri* parotoid gland poison has shown activity against different human cancer cells proliferation [13], activate human complement system [14] and inhibit chymotrypsin [15]. Protein components present anti-inflammatory, anti-nociceptive and toxic activities in mice [16] Similarly, protein from *Bufo bufo* parotoid secretions are likely to play a role in cardiotoxic effects [17] and *B. bufo, B. verrucosissimus* and *Bufotes variabilis* both parotoid and skin secretion protein are capable of inhibiting different gram-negative bacteria and cytotoxic effects on different types of cells [18].

Anuran cutaneous gland secretions are widely known to contain several classes of antimicrobial peptides (AMP) and function as the first barrier against microorganisms. Although frogs’ mucous gland secretions are broadly studied and there are more than 40 classes of AMP reported up to date [19], there is no survey regarding *R. schneideri* putative protein and AMPs in the cutaneous secretions. The “omics” technologies are powerful tools to overcome this issue.

Transcriptomics, one of the “omics techniques”, is one powerful approach to unravel peptides and protein in a holistic manner. Currently, RNA-seq is the state-of-art technique used to predict all protein molecules that can be produced by a specific issue with the greatest outcome of information, thus making possible the discovery of minor toxins that could not be detected through traditional techniques due to their low abundance in the final secretion [20]. This approach was used to unravel frogs AMP and adaptations [21, 22] and immune system [23]. However, there is no transcriptomic information regarding toads’ skin peptides and proteins.

In order to overcome this lack of information, we constructed a RNA-seq transcriptome from skin of an individual *R. schneideri* toad. The transcriptome was sequenced in duplicate using Illumina HiSeq 2500, the reads were treated and the contigs were de novo assembled with the aid of Trinity. The results were annotated against non-redundant (nr) NCBI database and enriched with database of anuran defense peptides (DADP). Thus, the cutaneous secretion from the same toad, milked prior its death, was used to carry out biochemical analysis, as assessing its protein profile by SDS-PAGE, RP-FPLC fractionation in C18 column, peptide and protein sequencing by Edman’s degradation and activity upon casein to better investigate this secretion. To the best of our knowledge, this is the first study to unravel the potential of *R. schneideri* cutaneous gland secretions.

## Methods

### Ethics statement

Animal’s experiments were designed according to the Normative Resolution N. 13, from Brazilian Minister of Science, Technology and Innovation. The experiments were reviewed and approved by the Animals usage Ethic Committee from School of Pharmaceuticals Science of Ribeirão Preto – University of São Paulo (N°: 15.1.341.60.2).

### Sample collection and RNA extraction

One adult *Rhinella schneideri* toad was sacrificed through anesthetic overdose and the skin was dissected for mRNA assessment. According to the animal’s body weight, a dose of combined anesthetic ketamine (10 mg/kg) and pentobarbital (150 mg/kg) was applied intraperitoneally after 3 days of cutaneous secretion milking through electric stimulation (5 V, 100 Hz, 140 ms). Toad skin patches were carefully dissected and washed with RNAlater® (Life technologies, USA), immediately thawed with dry ice and ethanol bath and kept at − 80 °C until the moment of use. All the instruments and materials used were previously cleaned in Diethylpyrocarbonate (DEPC) solution 0, 1% (*v*/v).

The total RNA was extracted using liquid nitrogen and TRIzol® reagent (Life technologies) following the manufacturer’s instructions. RNA integrity was assessed with 1% agarose gel and quantified with a Qubit® RNA assay Kit with a Qubit® 2.0 Fluorometer (Life technologies). Thus, the RNA integrity was attested using 2100 Bioanalyzer (Agilent, USA) analysis.

Toad’s cutaneous secretion (CS) was stored at − 20 °C until the moment of usage for RP-FPLC and biochemical analysis.

### Transcriptome construction and sequencing

The transcriptome was constructed using the TruSeq Stranded mRNA library kit (Illumina, USA) according to the manufacturer’s instructions. The library containing 100 bp fragments was paired-end sequenced in duplicate in the Illumina HiSeq 2500 platform (Illumina).

### De novo assembling and functional annotation

Raw reads were trimmed using FastQC (Q < 20) [24] and the adaptor sequences were discarded. The quality control was confirmed using the FastQC tool and the reads with good quality were submitted to the de novo assembling using the Trinity software with K-mer = 31. The reads were mapped against the constructed transcriptome using the Tophat tool to identify splice junctions between exons. Transcripts per million (TPM) was calculated using Salmon tool. The contigs were assembled against National Center for Biotechnology Information (NCBI) non-redundant (NR) database, with the aid of FunctionAnnotator website available at http://163.25.92.60/index.php [25], and specific anuran antimicrobial peptides (AMP) database DADP [26] using the blastx algorithm, to obtain the functional annotation. The resulting annotated sequences were the ones with cut-off value of significance lower than 1 × 10^− 5^, coverage higher than 70% and protein identity (pident) higher than 60.

### Fractionation of cutaneous secretion (CS) by RP-FPLC, SDS-PAGE and N-terminal sequencing

CS was lyophilized and the dried secretion (25 mg) was dispersed in deionized water (5 mL). The insoluble part was separated after centrifugation (10.000 x g, 5 min, room temperature) and the supernatant was filtered in 0.22 μm polyvinylidene fluoride (PVDF) membrane. CS solution (1,5 mL) was submitted to fast protein liquid chromatography (FPLC) in a C18 column (5 μm, 250 × 10.0 mm, 300 Å, Jupiter, Phenomenex) using Äkta Pure system (GE Healthcare) as described by Shibao et al. [15]. C18 column was firstly equilibrated with solution A (TFA 0, 1%) and the fractions were eluted with segmented gradient of acetonitrile until 100% of solution B (acetonitrile 60% in TFA 0, 1%) under 5 mL/min flow rate and 214 nm monitoring. The resulting fractions were collated and storage at − 20 °C until the moment of usage. The chromatographic profile was generated using the software Unicorn 5.20 (GE Healthcare).

An aliquot of 100 μL of each fraction was dried and dispersed in 50% acetonitrile (ACN) solution. Each fraction was submitted to sodium dodecyl-sulphate-polyacrylamide gel electrophoresis (SDS-PAGE), according to Schagger and Von Jagow [27]. In addition, different volumes (5, 10 and 20 μL) of the crude secretion used to RP-FPLC was also submitted to SDS-PAGE. Bench marker Amersham low molecular weight calibration kit for SDS electrophoresis (GE Healthcare) was also used to estimate protein molecular weight. The gel was submitted to 90 V, 40 mA and 15 W for 4 h and stained with the PlusOne Silver Staining Kit (GE Healthcare).

Protein fractions identified in the SDS-PAGE were submitted to amino terminal sequencing through Edman degradation [28] by the automatic protein sequenator model PPSQ-334 (Shimadzu).

### Peptides and protein alignment

Primary peptides and proteins sequences were deduced from the cDNAs sequences from the transcriptomes with Expasy translator tool. The deduced sequences and the sequences determined by N-terminal sequencing were aligned using Multalin algorithm [29]. The alignments were formatted using Espript 3.0 [30].

### Caseinolytic activity

A chromogenic proteolytic assay with the CS was performed in the presence and absence of ethylenediamine tetraacetic acid (EDTA) and phenylmethylsulfonyl fluoride (PMSF). The assay was conducted following the method described by Wang [31]. For this assay, we used 90 μL azocasein (10 mg/mL) in 50 mM Tris-HCl buffer with 0.15 M NaCl and 0.15 M CaCl_2_ (pH 8.0), different volumes (10 μl, 20 μl and 30 μl) of CS (5 mg of dried secretion in 1 mL of deionized water), 100 mM EDTA or 100 mM PMSF and Tris-HCl buffer solution (100 mM) to complete the reactions to 120 μL. Positive control was performed using 10 μL Trypsin (100 mM) and negative control was carried out using the same volume of buffer. The reactions were incubated at 37 °C for 90 min and stopped by adding 120 μL of 0.5 M trichloroacetic acid. All the tubes were centrifuged at 1000 *x g* for 5 min, 150 μL of the supernatant was mixed with the same volume of 0.5 M NaOH and absorbance was determined at 450 nm. This assay was carried out in triplicate. Data were plotted using the software GraphPad Prism 6.0 (GraphPad Software Inc).

## Results

### Transcriptome sequencing, de novo assembly and functional annotation

The same transcriptome was sequenced in duplicate resulting in 129,467,414 and 131,652,320 raw reads (considering forward and reverse reads) for each duplicate. The data obtained from de novo assembling is summarized in Table 1. The contigs were analyzed according to their functional annotation regarding Gene Ontology (Additional file 1), hits with deposited nucleotide and protein sequences from nr NCBI database and DADP, being the latter very important for results enrichment, once there is not much information regarding toads in NCBI database.

**Table 1:** Statistical analysis of the transcriptome sequencing and de novo assembling with Trinity

Parameters	Values
Number os trimmed reads	174,308
Average size (bp)	633.58
N50 (bp)	365
Largest sequence (bp)	22,684
Smallest sequence (bp)	201

### AMP assessment

The functional analysis of the transcriptome data and the AMP Database showed the presence of 43 different peptides and protein classes. Table 2 summarizes the more abundant contigs (TPM > 100) and are clustered in 33 classes of AMP. The five major classes of AMP, considering the TPM values, are kassinin, temporin, peroniin, rugosauperolein and buforin.

**Table 2:** List of main antimicrobial peptides from transcriptome

AMP families	Accesion number	Identification	ENA Transcript identification	TPM
Aurein	sp|P69021	Aurein-3.1	TRINITY_DN64440_c0_g1_i1	256.721
Bombesin	sp|P84211	Bombesin-like peptide	TRINITY_DN48099_c0_g1_i1	164.379
	sp|P84212	Bombesin-like peptide	TRINITY_DN27392_c0_g1_i1	192.298
			TRINITY_DN61842_c0_g2_i1	106.099
			TRINITY_DN80913_c0_g1_i2	102.443
	sp|P84214	Bombesin	TRINITY_DN71876_c0_g1_i1	117.297
	sp|P86026	[Asn3,Lys6,Phe13]3–14-bombesin	TRINITY_DN76333_c0_g1_i1	135.698
Bradykinin-like peptide	sp|P84823	Bradykinin-like peptide	TRINITY_DN71047_c0_g1_i2	123.521
Brevinin	sp|P32423	Brevinin-1	TRINITY_DN70354_c0_g3_i1	175.329
	sp|P82233	Brevinin-1Ta	TRINITY_DN73322_c2_g2_i1	122.993
Buforin	sp|C0HJB7	Buforin-EC	TRINITY_DN54614_c0_g1_i1	766.952
			TRINITY_DN60267_c0_g1_i1	190.875
			TRINITY_DN60267_c1_g1_i1	231.457
			TRINITY_DN60267_c0_g2_i1	228.282
Caerin	sp|P69032	Caerin-2.3	TRINITY_DN66726_c0_g1_i1	182.654
			TRINITY_DN66726_c0_g1_i2	121.242
Caeridin	sp|P82076	Caeridin-4	TRINITY_DN79589_c4_g3_i1	226.024
Citropin	sp|P81840	Citropin-1.2	TRINITY_DN86384_c3_g3_i1	111.715
Cruzioseptin	sp|C0HK11	Cruzioseptin-15	TRINITY_DN70275_c0_g1_i1	293.883
Dermaseptin	sp|P85523	Dermaseptin-1	TRINITY_DN76697_c0_g1_i2	115.602
Hylambatin	sp|P08614	Hylambatin	TRINITY_DN87362_c21_g12_i1	174.475
Hylin	sp|P84003	Hylin-b2	TRINITY_DN84946_c0_g1_i2	971.000
Hyposin	sp|P84957	Hyposin-HA3	TRINITY_DN72338_c1_g1_i1	616.344
Japonicin	sp|P83306	Japonicin-1	TRINITY_DN64014_c0_g1_i1	310.058
Kassinin	sp|P08611	Kassinin	TRINITY_DN120600_c0_g1_i1	346.986
			TRINITY_DN127618_c2_g1_i1	5081.92
			TRINITY_DN52216_c0_g1_i1	247.853
	sp|P42988	Kassinin-like peptide	TRINITY_DN81065_c1_g3_i1	130.979
			TRINITY_DN87139_c3_g3_i1	164.946
Nigrocin	sp|B3A0M7	Nigrocin-2JDb	TRINITY_DN87453_c9_g4_i1	327.271
	sp|P0C8U1	Nigrocin-2HSb	TRINITY_DN85182_c2_g10_i1	122.641
			TRINITY_DN85182_c2_g3_i1	221.000
Peptide PGLa	sp|C0HKP4	Peptide PGLa-R6	TRINITY_DN80328_c3_g2_i1	229.068
Peroniin	sp|P86487	Peroniin-1.1	TRINITY_DN53498_c0_g1_i1	279.654
	sp|P86488	Peroniin-1.1a	TRINITY_DN67234_c0_g1_i1	347.803
	sp|P86493	Peroniin-1.3a	TRINITY_DN81622_c0_g1_i1	129.528
	sp|P86495	Peroniin-1.1b	TRINITY_DN46821_c0_g1_i1	841.554
Phyllocaerulein	sp|P86625	[Arg4]-Phyllocaerulein	TRINITY_DN85513_c6_g2_i2	280.537
Phylloseptin	sp|P84569	Phylloseptin-4	TRINITY_DN87185_c0_g1_i2	109.815
	sp|P84931	Phylloseptin-3	TRINITY_DN64642_c0_g2_i1	168.944
	sp|P86283	Phylloseptin Bu-2	TRINITY_DN78083_c0_g4_i1	389.329
			TRINITY_DN78083_c0_g6_i1	334.985
			TRINITY_DN78083_c0_g8_i1	126.679
Ranacyclin	sp|P83663	Ranacyclin-E	TRINITY_DN46765_c0_g1_i1	685.023
			TRINITY_DN81581_c5_g1_i4	371.815
Ranalexin	sp|P82876	Ranalexin-1Ca	TRINITY_DN110865_c1_g1_i1	343.086
Ranatensin	sp|P08951	Ranatensin-C	TRINITY_DN70922_c0_g2_i1	116.215
Ranauterin	sp|P86020	Ranatuerin-9	TRINITY_DN85516_c7_g13_i1	111.483
Riparin	sp|P86125	Riparin-1.2	TRINITY_DN83324_c7_g3_i1	215.018
			TRINITY_DN92795_c0_g1_i1	164.545
	sp|P86126	Riparin-1.3	TRINITY_DN85374_c2_g1_i1	330.835
			TRINITY_DN86546_c0_g1_i2	264.226
			TRINITY_DN86547_c3_g6_i1	130.999
Rothein	sp|P86510	Rothein 2.2	TRINITY_DN87840_c8_g1_i1	261.426
Rugosin	sp|P08616	Rugosauperolein-2	TRINITY_DN87822_c5_g12_i1	826.816
	sp|P84912	Rugosin A-like peptide	TRINITY_DN82274_c0_g1_i5	196.425
			TRINITY_DN82274_c0_g1_i6	314.015
Signiferin	sp|P86123	Signiferin-1	TRINITY_DN86818_c0_g1_i1	140.473
Temporin	sp|C0HJB9	Temporin-ECa	TRINITY_DN86407_c15_g1_i1	274.636
			TRINITY_DN86407_c15_g2_i1	915.026
			TRINITY_DN86407_c15_g3_i1	478.761
			TRINITY_DN86407_c15_g4_i1	527.791
			TRINITY_DN86407_c15_g5_i1	256.836
			TRINITY_DN86407_c15_g7_i1	176.844
	sp|P0C5X6	Temporin-1DYa	TRINITY_DN29229_c0_g1_i1	133.681
	sp|P56917	Temporin-A	TRINITY_DN13166_c0_g1_i1	527.935
			TRINITY_DN13166_c0_g2_i1	158.363
	sp|P82832	Temporin-1Lc	TRINITY_DN2695_c0_g1_i1	175.444
			TRINITY_DN72771_c0_g1_i1	105.843
			TRINITY_DN73309_c0_g1_i1	140.262
			TRINITY_DN80955_c1_g1_i1	254.369
			TRINITY_DN80955_c1_g2_i1	387.789
			TRINITY_DN84769_c0_g1_i1	373.663
			TRINITY_DN86165_c3_g1_i1	155.493
	sp|P82881	Temporin-1Cb	TRINITY_DN56332_c0_g1_i1	105.694
	sp|P84858	Temporin-GH	TRINITY_DN77159_c0_g2_i9	102.951
Tigerinin	sp|P82652	Tigerinin-2	TRINITY_DN70574_c0_g1_i1	242.086
			TRINITY_DN71694_c0_g1_i1	101.217
			TRINITY_DN85395_c7_g6_i1	130.962
	sp|C0HL42	Tigerinin-2OS	TRINITY_DN85615_c2_g3_i2	125.931
	sp|P82651	Tigerinin-1OS	TRINITY_DN17276_c0_g1_i1	245.073
			TRINITY_DN43562_c0_g1_i1	104.073
			TRINITY_DN6203_c0_g1_i1	454.682
			TRINITY_DN73092_c0_g1_i1	248.482
			TRINITY_DN85479_c6_g29_i1	457.183
Uperolein	sp|P08612	Uperolein	TRINITY_DN111770_c0_g1_i1	107.555
			TRINITY_DN83356_c0_g1_i1	424.761
			TRINITY_DN83356_c0_g1_i2	339.468
			TRINITY_DN83356_c0_g1_i3	101.171
			TRINITY_DN81958_c0_g1_i1	277.617
			TRINITY_DN81958_c0_g1_i2	216.205
Uperin	sp|P82036	Uperin-5.1	TRINITY_DN87247_c0_g1_i1	332.281
			TRINITY_DN87247_c1_g1_i1	137.665
			TRINITY_DN87247_c0_g1_i3	185.119
			TRINITY_DN77327_c0_g2_i1	238.182
			TRINITY_DN87210_c0_g1_i1	105.306
			TRINITY_DN87210_c0_g2_i1	132.473

### Other proteins of interest

The main protein of interest that are not considered AMP are listed in Table 3. Two contigs related to cobatoxin were found in this study. The first one, identified as TRINITY_DN69643_c0_g1_i, is identical to cobatoxin from *Helicoverpa armigera,* identified by access number ADR51150.1 (gi|313,247,974). The second one, identified as TRINITY_DN121110_c0_g1_i1, has matched cobatoxin A from *Spodoptera exigua* (gi|827,029,657).

**Table 3:** List of other peptides and protein of interest from the transcriptome

Protein class	Accession number	Identification	ENA transcript identification	TPM
Cobatoxin	gb|DR51150.1	Cobatoxin	TRINITY_DN69643_c0_g1_i1	94.407
	gb|AKJ54497.1	Cobatoxin A	TRINITY_DN121110_c0_g1_i1	0.40738
Galectin	gb|KPJ04718.1	Galectin-4	TRINITY_DN74940_c0_g1_i1	0.372625
	gi| 847,127,031	Galectin-8	TRINITY_DN78589_c0_g2_i1	149.955
	gi|768,932,680	Galectin-4-like	TRINITY_DN78656_c0_g1_i1	224.999
	gi|692,190,428	Galectin 9	TRINITY_DN80231_c0_g1_i1	0.180761
			TRINITY_DN80231_c0_g1_i2	0.188734
	gi|591,365,832	Galectin-9C-like	TRINITY_DN83688_c1_g1_i1	101.232
			TRINITY_DN83688_c1_g1_i4	170.619
	gi|928,062,140	Galectin-4-like	TRINITY_DN83688_c1_g1_i3	0.702454
	gi|512,835,424	Galectin-12	TRINITY_DN86454_c0_g1_i1	197.028
	gi|512,835,420	Galectin-12	TRINITY_DN86454_c0_g1_i2	189.779
			TRINITY_DN86454_c0_g1_i3	0.440812
			TRINITY_DN86454_c0_g1_i4	0.561889
	gi|2,554,855	Galectin A Chain A,	TRINITY_DN92920_c1_g1_i1	672.677
Ficolin	gi|512,864,759	Ficolin-2-like	TRINITY_DN75601_c0_g1_i1	0.832349
	gi|530,624,987	Veficolin-1-like	TRINITY_DN81241_c1_g5_i1	369.808
	gi|611,978,444	Ficolin-2-like	TRINITY_DN85932_c0_g1_i3	104.045
	gi|512,864,763	Ficolin-2-like	TRINITY_DN86611_c9_g1_i1	932.407
	gi|148,230,483	Ficolin	TRINITY_DN86611_c9_g2_i1	120.763
			TRINITY_DN86611_c9_g2_i2	205.043
Phospholipase A_2_	gi|512,862,492	Cytosolic Phospholipase A2 zeta	TRINITY_DN47903_c0_g1_i1	0.447251
	gi|512,929,364	Group XIIA secretory phospholipase A2	TRINITY_DN86978_c1_g1_i1	0.340838
	gi|530,606,756	Phospholipase A2, minor isoenzyme-like	TRINITY_DN69641_c0_g1_i1	0.724376
	gi|657,561,888	Group 10 secretory phospholipase A2-like	TRINITY_DN71078_c0_g1_i1	111.032
	gi|700,363,984	Cytosolic phospholipase A2 gamma	TRINITY_DN84494_c4_g4_i1	0.294445
			TRINITY_DN84494_c4_g5_i1	0.141515
	gi|847,119,410	85/88 kDa calcium-independent phospholipase A2	TRINITY_DN79625_c2_g1_i1	0.48039
			TRINITY_DN79625_c2_g1_i2	0.508773
	gi|884,758,924	Cytosolic phospholipase A2 gamma	TRINITY_DN60871_c0_g1_i1	0.0554201
			TRINITY_DN60871_c0_g2_i1	0.273762
			TRINITY_DN60871_c0_g3_i1	0.081637
			TRINITY_DN60871_c0_g4_i1	0.278435
	gi|512,862,492	Cytosolic phospholipase A2 zeta	TRINITY_DN54445_c0_g1_i1	162.823
	gi|148,223,595	Cytosolic phospholipase A2	TRINITY_DN71015_c0_g1_i1	107.048
			TRINITY_DN71015_c0_g2_i1	0.0297482
	gi|48,429,036	Phospholipase A2 crotoxin basic subunit CBc	TRINITY_DN72825_c0_g1_i1	320.644
	gi|129,456	Phospholipase A2 homolog crotoxin acid subunit CA	TRINITY_DN72825_c0_g2_i1	388.741
	gi|512,834,221	Group XV phospholipase A2 isoform X2	TRINITY_DN74674_c0_g1_i1	202.101
	gi|163,916,015	Phospholipase A2, group XIIA	TRINITY_DN74706_c0_g1_i1	250.869
Metallo proteases	gi|148,222,675	Disintegrin and metalloproteinase domain-containing protein 22 precursor	TRINITY_DN11900_c0_g1_i1	0.414536
Serine proteases	gb|ACI32835.1	Serine proteinase-like protein 1	TRINITY_DN79566_c0_g1_i1	0.486137
	gi|913,306,165	Serine proteinase stubble	TRINITY_DN86427_c0_g1_i1	0.326702
	gb|ACI32835.1	Serine proteinase-like protein 1	TRINITY_DN87507_c0_g1_i1	17.516
			TRINITY_DN87507_c0_g1_i3	126.092
			TRINITY_DN87507_c0_g1_i4	156.004
			TRINITY_DN87507_c0_g1_i5	0.772103
	gi|380,875,411	Thrombin-like Enzyme gyroxin B1.4	TRINITY_DN87578_c1_g1_i2	0.172693
			TRINITY_DN87578_c1_g1_i5	0.125578
			TRINITY_DN87578_c1_g1_i6	0.658672
	gb|AEJ32000.1	Serine proteinase 6	TRINITY_DN87578_c1_g1_i3	0.266027
			TRINITY_DN87578_c1_g1_i4	0.246148
			TRINITY_DN87578_c1_g1_i8	0.29959
	gi|827,563,139	Serine proteinase-like protein isoform X1	TRINITY_DN87890_c1_g6_i3	0.193263
	gb|AAM69353.1	Serine proteinase-like protein 2	TRINITY_DN87890_c1_g6_i5	0.508857

**Fig. 1:**
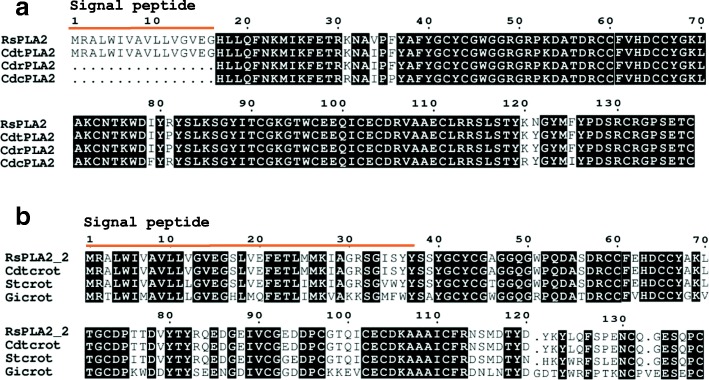
Sequence alignment of phospholipases A_2_ discovered in this transcriptome (RsPLA_2_) and those available in databases. **a** Alignment of protein codified by contig TRINITY_DN72825_c0_g1_i2, named RsPLA2 and different basic crotoxins subunits from *Crotalus durissus terrificus* CdtPLA_2_ (PA2B6_CRODO), *Crotalus durissus collilineatus* CdcPLA_2_ (PA2B6_CRODO) and *Crotalus durissus ruruima* (PA2BA_CRODR). **b** Alignment of protein codified by contig TRINITY_DN72825_c0_g1_i1, named RsPLA2_2 and different acidic crotoxins subunits, also known as crotapotin, from *Crotalus durissus terrificus* Cdtcrot (PA1A_CRODU), *Sistrurus tergeminus* Stcrot (PA2A_SISTE) and *Gloydius intermedius* Gicrot (A0A096XPP1_GLOIT). Signal peptide is indicated by the orange line above the sequences. Alignment was generated with the aid of Multalin and formatted using Espript 3.0

**Fig. 2:**
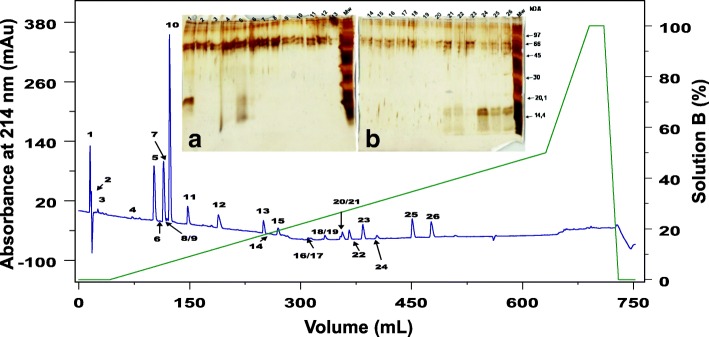
Cutaneous secretion chromatogram in C18 column RP-FPLC and the SDS-PAGE profile of each fraction. The blue line represents the absorbance monitored at 214 nm and the green line represents the concentration of solution B. Each fraction was analyzed in SDS-PAGE stained with silver (insert figures). Insert figure a represents fractions CS1 to CS13 and insert figure b shows fractions CS14 to CS26. The wells at left show the low molecular weight marker from GE Healthcare

In the present transcriptome we found 19 full-length sequences with high homology to PLA_2_. Interestingly, two contigs are similar to snake PLA_2_. Contig TRINITY_DN72825_c0_g1_i1 encodes a PLA_2_ highly similar to *Crotalus durissus terrificus* PLA_2_ crotoxin basic subunit (gi 48,429,036) (Fig. 1a). This PLA_2_ was also identified in the raw skin secretion in the fractions 24A, 25 and 26 (Fig. 2) and confirmed by Edman degradation sequencing of the fractions. Contig TRINITY_DN72825_c0_g2_i1 is also related to PLA_2_ from *C. d. terrificus* (Fig. 1b), but to the acid subunit (gi|129,456).

**Fig. 3:**
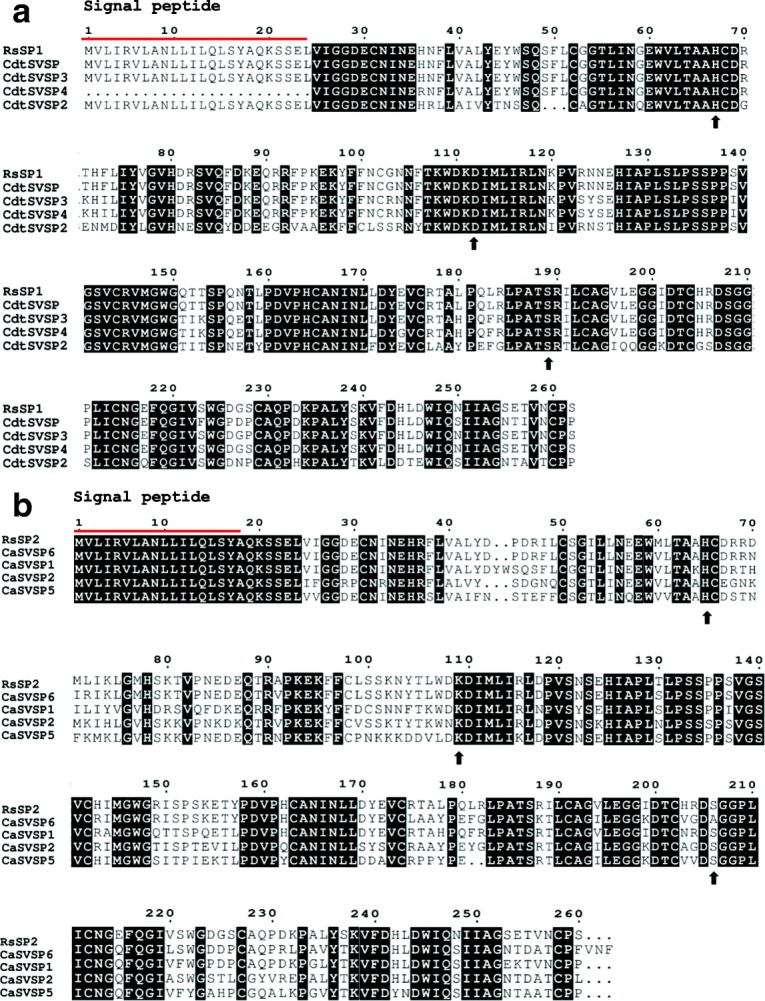
Sequence alignment of serine protease discovered in this transcriptome (RsSP) and those from different snake venoms (SVSP) available in datadases. **a** Alignment of protein codified by contigs TRINITY_DN87578_c1_g1_i3, TRINITY_DN87578_c1_g1_i4 and TRINITY_DN87578_c1_g1_i8, named RsSP1 and gyroxin serine proteases from snake venoms (SVSPs) from *Crotalus durissus terrificus* (CdtSVSP3, VSP13_CRODU), CdtSVSP 2 (VSP21_CRODU) CdtSVSP4 (VSP14_CRODU). **b** Alignment of serine protease codified by contigs TRINITY_DN87578_c1_g1_i2, TRINITY_DN87578_c1_g1_i5 and TRINITY_DN87578_c1_g1_i6, named RsSP2 and gyroxin SVSP from *Crotalus adamanteus* 1, CaSVSP1 (VSP1_CROAD), CaSVSP2 (VSP2_CROAD), CaSVSP5 (VSPE_CROAD) and CaSVSP6 (A0A1W7RB84_CROAD). Signal peptide is indicated above the sequences and the arrows beside the alignment indicates the amino acids that are important to the catalytic activity. Alignment was generated with the aid of Multalin and formatted using Espript 3.0

This study identified one full-length contig related to metalloproteases and 14 contigs related to serine proteases. Contig TRINITY_DN11900_c0_g1_i1 is highly homologous to a metallo-disintegrin from *Xenopus laevis.* Fourteen full-length sequences related to serine proteases were obtained. Six of them showed high similarity to *Crotalus ssp* snakes, being three (TRINITY_DN87578_c1_g1_i2, TRINITY_DN87578_c1_g1_i5, TRINITY_DN87578_c1_g1_i6,) containing the same coding sequence (herein named RsSVSP) highly related to gyroxin (Fig. 3). Contigs TRINITY_DN87578_c1_g1_i3, TRINITY_DN87578_c1_g1_i4 and TRINITY_DN87578_c1_g1_i8 encode a protein (RsSVSP2) very similar to serine protease 6 from *C. adamanteus* (gi|338,855,342).

Thirteen complete open reading frames (ORFs) related to galectins and 6 related to ficolins were found in the transcriptome. From those, 12 are related to predicted galectin from different genomes and transcriptomes. Contig TRINITY_DN92920_c1_g1_i1 is similar to galectin from *Rhinella arenarum* ovary. Four complete ORFs were found matching different galectins from *Xenopus* genome assessment. All contigs related to ficolins were annotated against model organisms’ genome (*Xenopus spp.* and *Monodelphis domestica).*

### Fractionation of CS by RP-FPLC, SDS-PAGE and N-terminal sequencing

Crude secretion SDS-PAGE profile is shown in Additional file 2. CS was separated in 26 fractions, named CS1 to CS26 (Fig. 2). The fractions were further submitted to SDS-PAGE, gel was stained with silver and the fractions named CS1, CS24, CS25 and CS26 (Fig. 2, insert) were identified containing protein compounds. In addition, probably fractions CS5, CS21 and CS22 also contain protein molecules, but due to its low concentration, they were not investigated in this study.

Both gels show some interference on their top, probably caused by the silver staining. It is possible to see bands with approximately 16 kDa in the fractions CS24, CS25 and CS26, which showed similar diffusion profiles. Therefore, these fractions were submitted to N-terminal sequencing by Edmans’ degradation, but it was possible to obtain only CS1 and CS24 – CS26 partial sequences (Table 4).

**Table 4:** N-terminal sequences from the poison fractions by Edman’s degradation technique

Fraction	Protein sequence	Protein family
CS1	RWEECEDCDDDQDDQQQQQCAKDDDDE---Q-QQD	Lectin
CS24a	GLLEFNKMIKFETRKNAIPFYAFYGCYCGWGGRRRPK	PLA_2_
CS24b	-TPFYKGAAGQQVPQDIVNYYAFGGCQK—EP-PMRY-EY-VNAGGDQ-------D	Galectin
CS25	-L-EFNKMIKFETRKNAIPFYAFY	PLA_2_
CS26	-L-EFNKMIKFETRKNAIPFYAFYGCYCGWGGRRRPKDA	PLA_2_

### Caseinolytic activity

**Fig. 4:**
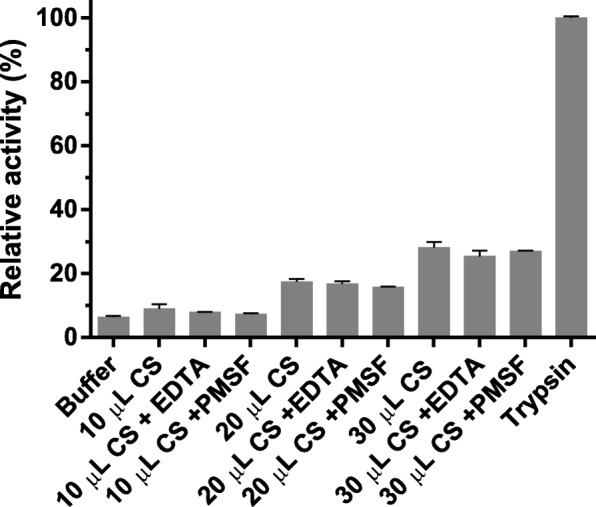
Caseinolytic activity assay. Azocasein degradation was determined spectrophotometrically. Different volumes of CS were incubated in presence of EDTA or PMSF. Buffer was used as negative control and Trypsin as positive control. The degradation was measured in triplicate and normalized to relative activity compared to Trypsin

The transcriptome functional annotation showed some sequences that can be related to serine and metalloproteases. In order to investigate if the sequences could really deduce these enzymes, we performed a proteolytic test using azocasein as substrate (Fig. 4). CS was capable of degrading azocasein. The tests in presence of EDTA and PMSF did not show differences.

## Discussion

Although toads are widely spread in Brazilian and Latin America territory, its cutaneous peptides and proteins have come to the spotlight only recently and the scarce information hinders its assessment [11, 16, 17].

The present study reveals the abundance of the *Rhinella schneideri* mucous gland defense peptides and protein through the RNA-seq transcriptome and some peptides and proteins present in its secretions. We used the cutaneous secretions from one specimen that was milked prior the gland extraction to obtain a more accurate result comparing the biochemical tests with the transcriptome.

Transcriptome survey is snapshots of mRNA obtained in a specific time. Therefore, the first step of this study was milking the toad’s skin secretions to maximize the transcripts production. We also used the secretion to carry out biochemical analysis subsequently. Using the secretions from the same toad to perform the experiments carried out in this paper allows us to discard any variation that may occur and obtain a more accurate analysis. RNA-seq analysis revealed the presence of distinct proteins and peptides. Most of the transcripts are related to housekeeping function, as expected, but we found novel proteins in the skin. As frog defense peptides are better characterized, we enriched the functional annotation using DADP database. Using this approach, there were several classes of AMP that are summarized in Table 2. The contigs that presented the highest values of TPM belong to tachykynins, temporin, peroniin and buforin classes. *Bufo bufo, B. verrucossismus* and *B. variabilis* skin secretions act as antimicrobial agent against *Escherichia fecalis* and *E. faecium* with lower minimum inhibitory concentration than ampicillin [18].

The molecular classes of AMPs found in this transcriptome are discussed below.

### Kassinin and Rugosauperolein

Kassinin and rugasoperolein AMPS belong to the tachykinin family of neuropeptides. Tachykinins are well present in amphibian secretions and higher organisms, acting as neurotransmitters and neuroprotective agents in the latter. They present a C-terminal conserved region Phe-X-Gly-Leu-Met, that is known for this family’s activity [32]. Regarding amphibians secretions, kassinin, a dodecapeptide, was the first tachykinin described from *Kassina senegalensis* methanol skin extract, which shows similar activity as substance P, physalaemin and eldosein, being able to stimulated salivary secretion, act as hypotensive agent and stimulate smooth muscle [33, 34]. Contig TRINITY_DN127618_c2_g1_i1 is very similar to the first kassinin ever identified.

Rugosauperolein was discovered in *Uperoleia rugosa* skin methanol extracts and was named after the tachykinin uperolein [35].

Tachykinins are widely studied mainly in higher organisms due their interesting neuroprotective activity. Despite it is known that they are present in amphibian skin, their role as AMP is poorly studied. As their role as neurotransmitters is well established, here we hypothesize they act not as a AMP, but as a host defense molecule that prevents the toad from predation along with micro molecules that are already known to play this role [36]. We use the same hypotheses to the presence of peroniin. Although we cannot discard the possibility of peroniin present any antimicrobial activity, we believe it is produced to prevent *R. schneideri* to be swallowed, once tachykinins are known to show neuroactivity being able to cause gut tissue contraction [37].

### Temporin

Temporins were discovered in a cDNA library from *Rana temporaria* skin and later obtained in *R. temporalis’* skin secretions. They are effective against gram positive *Bacillus megaterium* and *Escherichia coli* with different sensitivities. This AMP family comprises anionic, hydrophobic, small peptides (8–17 amino acids) that are folded in alpha-helices and which potency is related to the final net charge [38, 39]. Following its first discovery, temporins were also found in secretions of different species of the genus *Euphycits*, *Limnonectes*, *Hypsiboas*, *Amolops*, *Hylarana* and *Lithobates* [39]. Regarding their biotechnological applications, there are more than 20 deposited patents related to them, which varies from their pharmacological use as anti-HIV to obtaining of transgenic plant resistant to pathogens [39].

Deposited contig TRINITY_DN86407_c15_g2_i1 is similar to temporin-ECa, from *Euphlyctis cyanophlyctis*, the skittering frog. As other temporins, it shows activity against gram-positive bacteria *E. coli*, *K. pneumonia*, *Micrococcus luteus* and *Staphylococcus aureus*, and low hemolytic activity [40] .

### Peroniin

Peroniins discovery was made through mass spectrometry analysis of *Litoria peronii* skin secretions in winter and summer. Albeit they are considered AMP, there is no report of their test against neither gram positive nor gram negative bacteria. In fact, peronnins are the major component of *L. peronii* secretions both summer and winter and they possess activity over smooth muscle causing its contraction. There is only one report of peroniin up to date [41].

### Buforin

This AMP family was first discovered in *Bufo Bufo gargarizans* stomach. This family comprises small peptides (approximately 6.5 kDa) and are effective against several gram positive and negative bacteria [42]. They are derived from histone H2A and belong to the toads’ innate immune system. While buforin I is secreted in the stomach protecting the toad against pathogen ingested microorganisms and further binding to the mucosa biofilm enhancing its protection, buforin II does not have its mechanism of action fully elucidated, but it is hypothesized they bind to microorganism’s nucleic acid destroying it [43]. In this study, contig TRINITY_DN54614_c0_g1_i1 is related to buforin-EC, isolated from skin secretions of frog *Euphlyctis cyanophlyctis*, which has shown activity against *Staphyloccoccus aureus* and *Escherichia coli* [40]. We believe temporins and buforins, along with other AMPs, act as protection against microorganisms, due to the well-established activity of these molecules against gram positive and negative bacteria. The diversity of AMP found in the skin proves that glandular secretions work as a biochemical enriched barrier for the toads’ protection.

In addition to the AMPs many other protein components, which probably have relevant roles for frog defense, have been identified in the transcriptome, among them cobatoxins, PLA_2_, proteases, ficolin and galectins. These molecular classes are discussed below. Interestingly, ficolin was one of the major contigs found in the transcriptome survey.

### Cobatoxin

Cobatoxins were firstly reported in the *Centruroides noxius* scorpion poison as potassium channel-blocking toxins, belonging to the α-K-toxins, subfamily 9. They are moderate affinity blockers of K^+^ voltage-dependent channels Shaker and K_v_1.1 [44]. Cobatoxin from *Helicoverpa armigera* is mostly connected to the insect defense. Its level of expression has significantly raised after infection with both gram positive and negative bacteria [45]. Regarding *Spodopetra exigua* cobatoxin, gene expression analysis from the insect midgut after the exposition of the insect to *B. thuringiensis* toxins revealed an increase of the mRNA coding for this protein, indicating that it plays a fundamental role in the insect defense [46]. Furthermore, an analysis of *Galleria mellonella* challenged against *Micrococcus luteus* has shown that cobatoxin is likely to maximize the potential of other innate AMPs from the insect [47].

Here, we hypothesize that cobatoxin is also part of the immune system of the toad.

We also found other proteins of interest that are not catalogued as AMP with the aid of non-redundant NCBI database annotation:

### Phospholipase A_2_

Phospholipase A_2_ (PLA_2_) catalyze the hydrolysis of phospholipids in the sn2 position releasing arachidonic acid and lysophosphatidic acid, which are precursors of signaling molecules in immune response, inflammation, pain and cell regulatory processes [48–50]. They can be found in different tissues and organelles and are often small proteins (14–18 kDa), and their stability varies according to the number of disulfide bonds. Secreted PLA_2_s are one of the major components of Elapidae and Viperidae snake venoms [51]. In fact, the crotoxin was the first toxin isolated almost 100 years ago and its sequence has been determined for more than 30 years now, and the cloning was successfully obtained in the 80’s [52, 53].

In *Crotalus* snake venoms, crotoxin is composed of two non-covalently bound subunits (one acidic and one basic). The basic component (CB) is a catalytic active PLA_2_ whereas the acidic component (CA) is a PLA_2_ catalytically inactive responsible to direct CB towards specific sites that lead to neurotoxic actions [54–58].

In humans, secreted PLA_2_ group IIA can be found in tears and it is most likely to play a defensive role in eyes defense against gram-positive bacteria, but no response to gram-negative bacteria [59]. These molecules are also found in dromedary tears and showed activity against both gram positive and negative bacteria [60]. Thus, a PLA_2_ isolated from *Daboia Russelli* venom was able to strongly inhibit gram negative bacteria and also showed activity against gram positive bacteria isolated from human [61] . Therefore, we also assume the protective action in toad skin.

### Serine and metalloproteases

The contig TRINITY_DN11900_c0_g1_i1 is highly homologous to a metallo-disintegrin protease that has been related to *Xenopus laevis* reproduction [62] and neural crest development [63]. Although it was possible to obtain a full-length transcript in neural cells, the metallo-disintegrin did not have the catalytic domain, which indicates this protein acts as a transmembrane receptor [63].

Several full-length sequences were related to serine proteases. Three of them containing the same coding sequence (herein named RsSVSP) highly related to gyroxin, a non-lethal serine protease with neurotoxic effects that causes a neurological syndrome in mice known for the animal’s movements as rotation to a barrel roll [64]. The contigs TRINITY_DN87578_c1_g1_i3, TRINITY_DN87578_c1_g1_i4 and TRINITY_DN87578_c1_g1_i8 encode a protein, named RsSVSP2, very similar to serine protease 6 from *C. adamanteus* (gi|338,855,342), also found in a transcriptoma survey [65].

### Ficolin and galectin

Ficolins are a group of oligomeric lectin that present fibrinogen-like and collagen-like domains and possess a carbohydrate binding domain (CRD), being N-acetylglucosamine (GlcNAc) the carbohydrate that presents the major number of galectins specificity [66, 67]. They are capable of activating complement system via lectin pathway, and aggregate some bacteria enhancing phagocytosis showing their relevant role in organisms’ defense [68].

Galectins are lectins which major ligand is β-galactose-containing glycoconjugates and show their CRD conserved. They may bind to cell-surface and matrix glycans, being able to control intracellular signaling, and protein interactions dependent pathways [69]. Due to its action over neurological system, we believe it also plays a possible role as host defense peptides (HDP) and may provide protections against animal predation adding or even enhancing the symptoms of micro molecules present in parotoid glands that show neurological effects [10].

The contig TRINITY_DN92920_c1_g1_i1 is similar to galectin, an S-type lectin, from *Rhinella arenarum* ovary, probably playing a developmental regulatory role [70]. In contrast, *Xenopus* galectins from the animal’s skin are believed to act as HDP [71]. Recently, a galectin was found in parotoid secretion from *R. schneideri* using mass spectrometry de novo sequencing [11].

Crude secretion has shown a different profile from parotoid poison presented before [11, 12]. In the cutaneous secretion (Additional file 2) it is possible state there are a richness of protein content which molecular weight varies from high molecular mass (around 100 kDa) to low molecular mass (less than 14 kDa), but there is a strong band with approximately 60 kDa that is common to the profile presented in male and female parotoid secretions from Piaui, Brazil [11]. After analyzing the variation of mass, crude secretion was submitted to RP-FPLC to further investigation.

The RP-FPLC of the secretion resulted in 26 fractions, which were further analyzed by SDS-PAGE. The fractions CS1, CS24-CS26 were submitted to Edman’s degradation sequencing, because they showed protein bands on SDS-PAGE. Other fractions might contain micro molecules from poison and this method of chromatography was already used for isolation of those molecules from *R. schneideri* parotoid poison before [15].

Fractions CS24, CS25 and CS26 showed sequences regarding a PLA_2_ from snake venom [72]. Fractions CS24a, CS25 and CS26 contain PLA_2_ isoforms similar to a basic PLA_2_ isolated from *Crotalus durissus terrificus* (PA2BF_CRODU). As expected, we were able to predict the protein sequence from the contig TRINITY_DN72825_c0_g1_i1. We also retrieved contigs related to the acidic PLA_2._ In crotoxin, one of the major components of *Crotalus spp.* venom, both units (basic and acidic PLA_2_) are bonded by a non-covalent bound. The acid subunit (also named crotapotin) is devoid of activity, acting as a stabilizer, and the basic subunit is catalytically active and toxic [52]. Despite its role in the snake venom, we hypothesize this PLA_2_ is related to toad’s immune system, similarly to secreted PLA_2_ in human [59] and dromedary tears [60], especially facing the probability of the toad infection by amoeba.

CS24b sequencing also presents a protein that showed similarity to a galectin-1 from *Rhinella arenarum* (gi|255a855; sp.|P5621). It is a beta-galactosyl-binding lectin discovered in toad ovary and similar to the mammalian one that binds to different carbohydrates intra and extra-cellular, probably regulating developmental process in toads oocytes [70]. There are three contigs related to this protein, but none of them is complete. *Xenopus laevis* is known to secrete a lectin, along with other defense peptides, which plays a role not only in defense against pathogenic microorganisms, but also against predators and another unknown structural role [71].

CS1 sequenced revealed this fraction contains more than one protein; the first putative protein is similar to a C-type lectin isolated from *Helicoverpa armigera* (gi|385,202,653) that is up regulated in the presence of *Escherichia coli* injection, indicating it role as a defense protein [45], but it was not found in the transcriptome. CS1 is also similar to the lectin from *Naegleria gliberia* (gi|290,983,012), an amoeba that can be found in moist habitats and freshwater, that was unrevealed in its genome [73]. We could not retrieve any contigs related to this protein, which indicates a possible contamination of the toad skin by this microorganism. This contamination may explain the high levels of defense peptides and proteins found in the transcriptome and other fractions. It is known that toads might produce toxins that are specific against pathogens [74].

Beyond the AMP assessment and the discovery of other peptides and proteins of defense, we also retrieved some proteases. As the transcriptome showed some evidence of serine and metalloproteases, we decided to carry out an exploratory experiment to survey the presence of these proteins in the poison. Azocasein degradation assay is a classic test to determine the presence of either of these enzymes since proteolytic activity is evaluated. Raw secretion can result in dose dependent azocasein degradation. We used EDTA, which is a chelator agent that inhibits metalloproteases, and PMSF that inhibits serine proteases to investigate which class is most likely to be present in the raw secretion and cause the casein degradation. Despite our best efforts, it was not possible to determine which class of protease was more active, once the controls with PMSF and EDTA did not show any statistical relevance. However, we believe serine protease are the main responsible for this result, since there are more full-length contigs with higher TPM in the transcriptome and only one low expressed (TPM < 1) full-length contig coding for a metalloprotease. In addition, a serine protease was found in *Bufo bufo* parotoid secretion through a proteomic analysis [17], indicating it may be produced in this toad secretion too, but further characterization is needed.

Here we presented the first transcriptome survey from *R. schneideri* skin. As the results showed, this study paves the way for discovering new molecules besides characterizing an important secretion and the glands where they are produced.

## Conclusions

Although *Rhinella schneideri* toads are known to possess bioactive molecules in its secretion, it is still poorly studied when compared to other venomous and poisonous animals. Most of the studies comprises the parotoid micro molecules secretions in detriment of cutaneous secretion. This is the first study to use a high throughput RNA-seq technology to investigate *R. schneideri* cutaneous secretions and the first one to focus on defense peptides and proteins. Furthermore, using the milked secretion and skin from the same toad allowed an accurate analysis of protein expression, once individual variation was dismissed. The results obtained herein showed evidence of novel AMP and enzymes that need to be further explored.

## Additional files

Additional file 1: Gene ontology of Rhinella schneideri skin transcriptome. The Gene Ontology is divided in biological proccess, molecular function and cellular component. (DOCX 13 kb)

Additional file 2: Eletrophoretic profile of Rhinella schneideri’s cutaneous secretion. Different volumes (5, 10 and 20 µL) of CS were analyzed by 12,5 % SDS-PAGE and stained with Coomasie Blue PhastGel ™ R-350. MW-molecular weight marker; 5 µL- 5 µL of CS; 10 µL- 10 µL of CS; 20 µL – 20 µLof CS. All the samples were reduced in the presence of β-mercaptoethanol and boiled for 10 minutes befora application in the SDS-PAGE. (PPTX 189 kb)

## Abbreviations

ACN: Acetonitrile
AMP: Antimicrobial peptides
CS: Cutaneous secretion
DADP: Anuran antimicrobial peptides database
DEPC: Diethylpyrocarbonate
EDTA: Ethylenediamine tetraacetic acid
ENA: European Nucleotide Archive
GO: Gene ontology
HDP: Host defense peptides
NCBI: National Center for Biotechnology Information
pident: Protein identity
PLA_2_: Phospholipase A_2_
PMSF: Phenylmethylsulfonyl fluoride
PVDF: Polyvinylidene fluoride
RNA-seq: RNA sequencing
RP-FPLC: Reversed phase fast protein liquid chromatography
SDS-PAGE: Denaturing polyacrylamide electrophoresis gel
SVSP: Snake venom serine protease
TCA: Trichloroacetic acid
TFA: Acid trifluoracetic
TPM: Transcripts Per Kilobase Million
