# Supplementary material for: Deep sequencing analysis of toad Rhinella schneideri skin glands and partial biochemical characterization of its cutaneous secretion
Source: J Venom Anim Toxins Incl Trop Dis. 2018 Nov 29;24:36. doi: 10.1186/s40409-018-0173-8 (PMC6267030; doi:10.1186/s40409-018-0173-8)
Supplement: Supplementary file 1 — Gene ontology of Rhinella schneideri skin transcriptome. The Gene Ontology is divided in biological proccess, molecular function and cellular component. (DOCX 13 kb) [file 40409_2018_173_MOESM1_ESM.docx]

| Function | Number of reads | % |
| --- | --- | --- |
| Biological Process |  |  |
| Positive Regulation of biological process | 9,066 | 4 |
| Negative regulation of biological process | 9,224 | 4 |
| Signaling | 11,850 | 5 |
| Cellular component organization or biogenesis | 13,333 | 6 |
| Localization | 13,659 | 6 |
| Developmental process | 13,891 | 6 |
| Response to stimulus | 17,543 | 8 |
| Multicellular organismal process | 14,786 | 7 |
| Regulation of biological process | 22,786 | 10 |
| Biological regulation | 24,952 | 11 |
| Metabolic process | 36,023 | 16 |
| Cellular process | 38,573 | 17 |
| Cellular component | | |
| Extracellular region part | 4,454 | 3 |
| Membrane-enclosed lumen | 5,841 | 3 |
| Extracellular region | 5,889 | 3 |
| Protein-containing complex | 11,271 | 6 |
| Membrane part | 13,636 | 8 |
| Organelle part | 15,672 | 9 |
| Membrane | 18,543 | 12 |
| Organelle | 28,989 | 16 |
| Cell part | 35,789 | 20 |
| Cell | 35,793 | 20 |
| Molecular function |  |  |
| Catalytic activity | 25,710 | 40 |
| Binding | 38,2 | 60 |
