# Supplementary figures and images for: Deep sequencing analysis of toad Rhinella schneideri skin glands and partial biochemical characterization of its cutaneous secretion
Source: J Venom Anim Toxins Incl Trop Dis. 2018 Nov 29;24:36. doi: 10.1186/s40409-018-0173-8 (PMC6267030; doi:10.1186/s40409-018-0173-8)

## Slide 1
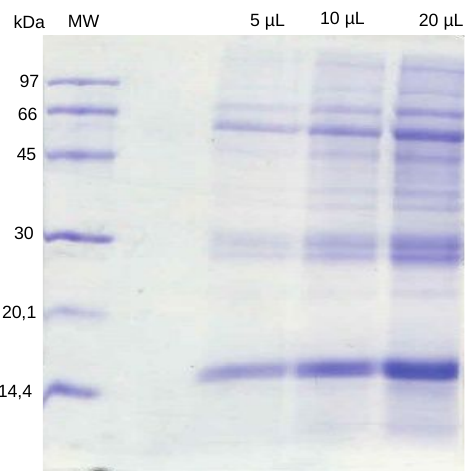

10 µL
20 µL
5 µL
MW
kDa
97
66
45
30
20,1
14,4

Supplement: Supplementary file 2 — Eletrophoretic profile of Rhinella schneideri’s cutaneous secretion. Different volumes (5, 10 and 20 µL) of CS were analyzed by 12,5 % SDS-PAGE and stained with Coomasie Blue PhastGel ™ R-350. MW-molecular weight marker; 5 µL- 5 µL of CS; 10 µL- 10 µL of CS; 20 µL – 20 µLof CS. All the samples were reduced in the presence of β-mercaptoethanol and boiled for 10 minutes befora application in the SDS-PAGE. (PPTX 189 kb) [file 40409_2018_173_MOESM2_ESM.pptx]
